# Health Aspects of Climate Change in Cities with Mediterranean Climate, and Local Adaptation Plans

**DOI:** 10.3390/ijerph13040438

**Published:** 2016-04-21

**Authors:** Shlomit Paz, Maya Negev, Alexandra Clermont, Manfred S. Green

**Affiliations:** 1 Department of Geography and Environmental Studies, University of Haifa, Haifa 3498838, Israel; 2 School of Public Health, University of Haifa, Haifa 3498838, Israel; maya.negev@gmail.com (M.N.); manfred.s.green@gmail.com (M.S.G.); 3 The Arava Institute for Environmental Studies, Kibbutz Ketura, D.N. Hevel Eilot 88840, Israel; alexclermont@gmail.com

**Keywords:** climate change, adaptation, cities, Mediterranean climate, climate action plans

## Abstract

Cities with a Mediterranean-type climate (Med-cities) are particularly susceptible to health risks from climate change since they are located in biogeographical hot-spots that experience some of the strongest effects of the changing climate. The study aims to highlight health impacts of climate change in Med-cities, analyze local climate adaptation plans and make adaptation policy recommendations for the Med-city level. We identified five Med-cities with a climate change adaptation plan: Adelaide, Barcelona, Cape Town, Los Angeles and Santiago. Beyond their similar Med-climate features (although Santiago’s are slightly different), the cities have different socio-economic characteristics in various aspects. We analyzed each plan according to how it addresses climate change-related drivers of health impacts among city dwellers. For each driver, we identified the types of policy adaptation tools that address it in the urban climate adaptation plans. The surveyed cities address most of the fundamental climate change-related drivers of risks to human health, including rising temperatures, flooding and drought, but the policy measures to reduce negative impacts vary across cities. We suggest recommendations for Med-cities in various aspects, depending on their local needs and vulnerability challenges: assessment of health risks, extreme events management and long-term adaptation, among others.

## 1. Introduction

### 1.1. Mediterranean Climate Type Regions and Their Vulnerability to Climate Change

The Mediterranean climate is characterized by a hot and dry season in summer and mild temperatures associated with rainfall in winter. Despite the apparent uniformity of the Mediterranean climate, there are differences between sub-areas, for example in the length of the dry season or a variance in precipitation amounts [1]. The Mediterranean climate type is found in five areas of the world including the Mediterranean Basin, the western United States (California) and Mexico (northwest Baja), central Chile, the Cape region of South Africa, and south and southwestern Australia [2]. These five areas are renowned for high levels of biological richness [3,4]. The mild Mediterranean climate and the proximity to the sea make it attractive to people, resulting in a disproportionately high conversion of ecosystems for agriculture, development, and other human uses. As a result, the Mediterranean climate regions are characterized by high population density and growth, as well as the extension of urban areas [5,6].

Since the Mediterranean climate is determined by the interaction between mid-latitude and sub-tropical circulation regimes and a complex morphology of mountain chains and land–sea contrasts, the Med-regions are vulnerable to climatic changes [7,8]. In fact, the Mediterranean ecosystems are among the most vulnerable to the global warming impacts [9]. Recent studies show that these changes have already been observed: for instance, since the 1960s, the Mediterranean basin has become warmer with a significant increase in the frequency, intensity and duration of heat waves. It is also characterized by a reduction in the availability of potable water as a result of a decrease in the total amount of precipitation, changes in rainfall patterns and water overuse by the growing population, mainly in urban areas [10,11]. In California the mean temperature has increased across all regions [12], and projected average temperatures are expected to rise dramatically in future decades, greatly exceeding the warming that has occurred to date since the late 19th century [13]. In recent years, California has experienced extreme drought [14]. According to Williams *et al.* [15] anthropogenic warming has intensified the recent droughts as part of a chronic drying trend that is becoming increasingly detectable and is projected to continue growing throughout the rest of this century. In the Western Cape region of South Africa significant warming trends were found and very warm days have become warmer or have occurred more regularly [16]. Under climate change projections, serious reductions in water supply and increases in the frequency of intense wildfires are expected for this region [17]. In southwestern Australia, the mean temperature has risen by more than 1 °C since 1910 and declines in rainfall have been observed in recent decades [18].

### 1.2. Climate Change Impacts on Health in Mediterranean Cities

Recent climatic changes and future projections have resulted in growing attention to the health effects on all human populations, both urban and rural [19]. These overall negative health effects impact in direct ways (e.g., heatwaves, flooding, and drought) and in indirect ways (e.g., food availability). The risks and the number of people exposed to them are influenced by social and economic development, technology, and health service provision. Consequently, urban areas where people, resources and infrastructure are concentrated are especially vulnerable to the health impacts of the changing climate [20,21].

Urban populations are increasing in absolute numbers and are also relative to rural populations in every part of the world. According to the UN [22], 54% of the world population lives in urban areas. Because cities concentrate populations, extreme weather events (such as severe heatwaves or flooding) affect a much larger number of people. Cities also concentrate poor populations who are especially vulnerable to the effects of climate change because of the conditions in which they live and the fact that they often lack adequate shelter or access to health services [23]. Another crucial factor is relating to the increasing proportion of the older population. This worldwide growing group is highly vulnerable (more than others) to health risks associated with extreme temperatures, infectious diseases and air pollution [19,21,24,25]. The population density in cities tends to lengthen the supply lines for essentials such as water, food, and energy sources. Extreme temperatures, storms or droughts that disrupt these urban lifelines can have serious consequences for the health of city residents [26]. In addition, the ways in which cities are constructed—reducing vegetation, covering large areas with impermeable surfaces, and obstructing natural drainage channels—make many city inhabitants more vulnerable to heatwaves, heavy precipitation, and other extreme weather events whose occurrence is found to be increasing [9,20]. The ways in which the changing climate affects the health of city inhabitants are detailed below:

- Extreme high air temperatures contribute directly to deaths from cardiovascular and respiratory disease, particularly among elderly people and those with chronic diseases. The impacts are worsened by the “urban heat island” effect, which results from greater heat retention of buildings and paved surfaces in urban areas.
- Direct physical injuries and deaths from extreme weather events such as intense rainfall, winds and floods. Extreme events may also damage homes, create dangerous transport conditions and disrupt the supply of medical and health services.
- Increasingly variable rainfall patterns (drought, floods) affect the supply of fresh water. A lack of safe water can compromise hygiene and increase the risk of water-borne diseases.
- Worsening air quality related to changes in temperature and precipitation resulting in the formation of smog may cause respiratory illnesses. High temperatures also raise the levels of ozone and other pollutants in the air that exacerbate cardiovascular and respiratory diseases. Pollen and other aeroallergen levels are also higher in extreme heat conditions.
- Illnesses and deaths occur from the spreading and expanded range of vector-borne infectious diseases.
- Food-borne diseases resulting from bacterial growth in foods exposed to higher temperatures.
- Unstable nutritional security among the urban poor who have reduced access to food (*i.e.*, shortages and rising prices) as a result of extreme events (extreme temperatures, drought, floods) [19,20,21].

In cities with a Mediterranean climate (Med-cities), people have to deal with cold in winter, which can be either dry or wet, and heat in summer, with high or low humidity. In the transitional seasons, over short periods of time, the climatic conditions can change from one extreme to another. Therefore, dealing with bioclimatic comfort and weather conditions in a Mediterranean climate may be more complicated than in more extreme environments [27]. These challenges are increasing since the Mediterranean climate-type regions are among the most vulnerable to the impacts of the changing climate [8]. In the Mediterranean basin itself, since many cities are old, they are compact and densely populated [28,29]. Furthermore, Med-cities often lack shading in public places, and when shading elements exist they are often designed in ways that do not provide sufficient shade during the hot hours, nor is their potential to reduce heat stress fulfilled [30]. Although air conditioning is essential in the long hot season, it is mainly used in areas with higher socio-economic standing while poor populations have less access to air conditioning [31]. In Med-cities, as a part of the local mentality, windows remain open for most of the hot months. Many activities, particularly social gatherings, occur in outdoor locations such as shaded balconies, courtyards, and outdoor restaurants [29]. These cultural behaviors increase the potential for contact with mosquitoes that may be vectors for infectious diseases. All the characteristics above make the residents of the Med-cities susceptible to the impacts of climate change.

### 1.3. The Importance of Adaptation Policy at the City Level

Climate change is a global phenomenon; and national governments have a crucial role to play in both mitigation and adaptation. Nevertheless, the local level is as important for protecting public health from this emerging threat: as was mentioned above, cities are vulnerable to climate change due to their population and infrastructure density. Med-cities are particularly susceptible since they are located at the hot-spots (areas of high biodiversity under threat from humans) of some of the strongest climate change effects [9].

Adaptation at the city level can prevent or significantly reduce vulnerability, including improvement of housing, building resilient infrastructure and strengthening the adaptation capacity of the general community and of low-income and vulnerable groups [32,33]. For example, modifications to vegetation cover and surface albedo can “offset projected increases in heat-related mortality by 40%–99%” ([34], p. 7). Indeed, implementation of a heatwave plan alongside public awareness and readiness of public health services were estimated to prevent 4000 cases of access mortality in France in 2006 [35]. Adaptation measures can have more than one health benefit. For example, improving the thermal efficiency of housing has two co-benefits: reducing electricity bills and reducing emissions of air pollutants thereby decreasing respiratory diseases. Also, increasing tree canopy cover has the co-benefits of reducing heat stress, and improving the living environment and one’s sense of wellbeing [36].

The current study aims to highlight health impacts of climate change in cities with Mediterranean climate, and to analyze in detail local climate adaptation plans in cities with Mediterranean climate-type (which are highly vulnerable to health risks as a result of the changing climate), in order to identify the climate change-related health impacts addressed in the plans, as well as the policy measures developed to adapt to these health impacts. Finally, adaptation policy recommendations for the city level in Mediterranean climate regions are proposed.

## 2. Methods

**Figure 1:**
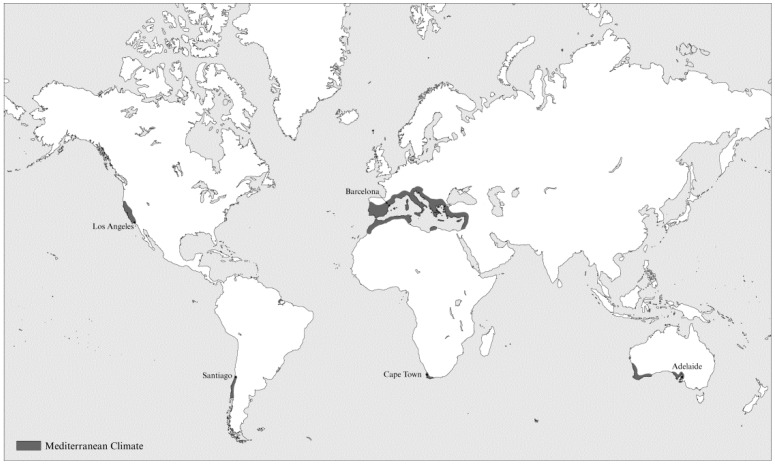
Location of Mediterranean climates and the five Med-cities in the current study.

We investigated different cities (20,000 < population < 4,000,000) with a Mediterranean climate that have climate action plans, using the following methods: internet search of municipal websites, search of keywords in the University of Haifa Library Catalogue, Google search engine, Google scholar and websites of international organizations such as the United Nations and World Health Organizations (keywords: adaptation, climate change plan, climate action plan, climate change adaptation plan, CAP, climate change in cities). We searched in English, French and Spanish. We identified five Med-cities [2,37] with a comprehensive plan which refers to various aspects of expected health impacts of climate change: Adelaide, Australia; Barcelona, Spain; Cape Town, South Africa; Los Angeles (LA), United States and Santiago, Chile (Figure 1). Each of these five large cities characterized by typical Mediterranean climate, serves as an example of one of the five Mediterranean-climate regions of the world [38]. These five examples deal with similar vulnerability challenges related to climate change and health in a Mediterranean climate (such as heat stress, floods, drought, loss of biodiversity) which may be more complicated than in more extreme environments [27]. It is important to note that the climate of Santiago is slightly different (a “continental Mediterranean climate”) since it is not a coastal city but located about 100 km from the South Pacific Ocean at an elevation of ~520 m.

While these five cities published climate action plans, to the best of our knowledge, many other major Mediterranean climate cities, particularly in the Mediterranean basin, did not have such plans.

We analyzed each plan according to the climate change-related drivers of health impacts among city dwellers in the First Assessment Report of the Urban Climate Change Research Network [20]:

1. Temperature extremes
2. Wind, storms, and floods
3. Fresh water supply and quality
4. Air quality and aeroallergens
5. Vector-borne diseases

To this list, we added three policy aspects that the First Assessment Report identified as important for health-related urban adaptation:

6. Protection of urban biodiversity and functioning ecosystems
7. Risks to vulnerable populations
8. Education and raising awareness.

For each of the above health drivers and policy aspects, we examined whether it was identified as a local risk in municipal and national vulnerability assessments. We then identified the types of policy adaptation tools that address it in the urban climate adaptation plans. Since certain aspects of health adaptation, such as heat management and disease surveillance, are often not under the authority of municipalities, but rather the authority of local and national public health agencies, we searched for and analyzed also national adaptation plans and local health agencies adaptation plans, and added additional policy instruments found in these documents.

As for the limitations of this study, this is a desk study in which we surveyed municipal climate plans that were published online by municipalities. We did not have access to unpublished materials, and the plans were published between two to eight years ago. Moreover, it is likely that departments and officials take policy measures to adapt for health aspects of climate change at the municipal level beyond the measures detailed in the general climate plans of the municipality. It was also beyond the scope of this study to examine the impact on the adaptation policy of institutional mechanisms and decision-making processes, roles and capacities. This paper provides a preliminary mapping of health-related climate strategies at the municipal level in cities with Mediterranean climate, and identifies adaptation gaps in these strategic plans. It can serve as a basis for future work that will include empirical research in particular cities.

## 3. Results and Discussion

Table 1 presents descriptive information on the five selected Med-cities. All five cities are characterized by hot summers (mean maximum temperature above 26 °C) and moderate winters. As was noted above, Santiago is not a coastal city and therefore its climate is slightly different. Beyond the Med-climate features, the cities have different socio-economic characteristics in various aspects: the population size (from fewer than 22,000 in the city of Adelaide (not in Greater Adelaide which is much larger) to around 4 million in LA), the population distribution by age (21.6% of the population in Barcelona is above 65 years old compared to only 5.5% in Cape Town), the country economy ranking by the World Bank (#1 for the USA and #42 for Chile), or the Country Human Development Index (#2 for Australia and #118 for South Africa). These socio-economic differences are important parameters in the ability of each city to deal with the impacts of the changing climate on the health and security of the city population.

Table 2 presents the climate action plans in the five Med-cities, analyzed according to the expected climate change-related drivers and outcomes for urban health of city dwellers. The risks are listed in themes inspired by Barata *et al.* [20], indicating whether they are identified as local risks in vulnerability assessments. Notably, in all cities the following were identified as local risks: rising temperatures, heavy rainfall and flooding, and fresh water supply and quality. Air quality was identified as a risk by Barcelona, Cape Town and LA vulnerability assesments, and food-water and vector-borne diseases were identified as risks in all cities excluding Adelaide. Loss of biodiversity was not identified as a health risk per se, but all cities developed adaptation measures to protect ecosystems.

### 3.1. Rising Temperatures: Heat Waves, Heat Stress, Heat Island Effect

The major health impact included in all five plans was that of rising temperatures, particularly heatwaves, heat stress and the heat island effect (that exacerbates the impacts of the heatwaves). Barata *et al.* [20] identified three types of policy tools to adapt to heat-related threats to public health: (1) urban design, including improving surface cover to increase reflectivity, increasing urban tree canopy and generating air movement through urban corridors; (2) heatwave management; and (3) reducing emissions. Heat wave management appears in all cities, while urban design to reduce the predicted increase in temperature was found in the action plans of all cities except Cape Town. Nevertheless, while all the plans we surveyed presented measures of adaptation to the rising temperatures, the specific tools to this end differ between cities. Specifically, Adelaide included a comprehensive heatwave management plan, including emergency procedures and development of formalized heat strategies. Cape Town is considering a comprehensive “Heat-Health” action plan including monitoring, emergency medical services, public and professional awareness; Santiago included a monitoring system but lacked other components for heatwave management, and Barcelona plans to develop an emergency program, early warning systems and protecting workers from heat-related health risks and protecting workers from heat-related health risks.

Alongside heatwave management, most of the plans included urban design as another adaptation measure to reduce negative health impacts of extreme heat. Yet each city presented a different focus of urban design: Adelaide and LA emphasized increasing vegetation and tree canopy that provides shade and cooling temperatures and reduces the urban heat island effect. Barcelona focused on drafting adaptation criteria into urban development plans, while Santiago included green standards in new development projects and protection of ventilation corridors.

### 3.2. Heavy Rainfall and Flooding

Winds, storms and floods are another consequence of climate change with severe impacts on human security and health, which adaptation can decrease significantly. With regard to this issue, most plans detail adaptation measures, but each city focuses on different ones even though four of the five cities are coastal and face similar challenges regarding adaptation to flooding (as a result of intense rainfall or sea waves during storms) and rising sea levels. In the example of infrastructure, Adelaide emphasized green infrastructure, protection from sea level rise and storm discharge management. Santiago emphasized blue infrastructure, namely revitalizing existing water flow networks, while Barcelona chose to start with mapping flood risks, developing an action plan for flood zones and monitoring. Cape Town details flood management as well as adaptation to the risk of sea level rise including risk assessment, economic modelling and regulation of a coastal protection zone by-law.

### 3.3. Fresh Water Supply and Quality

All cities address this crucial climate change-related driver. Adelaide mentions continuation of an existing plan, while all the other cities identify reducing water consumption in order to prepare for water shortages, with LA and Cape Town specifying ambitious targets of reducing demand by 20%. Each city has somewhat different foci in its plan, but all identify the need for preserving water resources while facing increased scarcity.

### 3.4. Air Quality

Air quality is another important climate change-related driver, particularly of respiratory illness. Reducing greenhouse gas emissions is a predominant mitigation measure that decreases climate change and its health outcomes in the long-term; reducing air pollution also decreases the heat island effect and its impact on heat-related morbidity and mortality [21]. Of the climate plans we surveyed, LA, Cape Town and Barcelona were the cities that identified air quality as a climate change induced risk to public health. These cities included both mitigation and adaptation strategies, and these included reducing emissions. Barcelona also included urban designs to reduce air pollution and exposure to air pollution, by establishing car-free areas, for instance. Santiago, which did not identifiy air quality as a climate change induced risk, did include in its plan more green spaces to improve air quality. None of the plans referred to an increase in aeroallergens.

### 3.5. Water-Borne Diseases, Food-Borne Diseases, Vector-Borne Diseases

Vector-borne diseases (VBDs) are a real threat in several Mediterranean-climate regions, and this health risk is expected to increase due to climate change [84]. All cities identified this as a risk, except Adelaide. Vector-borne disease surveillance was mentioned in the plans of Barcelona, Cape Town (particularly malaria, noting that the disease is not a critical concern but that possible future spread should be noted) and LA. Cape Town also included adaptation to the possibility that rising temperatures will lead to an increase in water- and food-borne diseases and jeopardize food security. Cape Town was also the only city that mentioned food security and urban agriculture policy. Santiago is planning to develop capacities to address potential introduction of VBDs.

### 3.6. Loss of Biodiversity, Functioning Ecosystems

The loss of biodiversity will affect human health and decrease resilience to climate change. It may to lead to crop failure as well as to an increase in the transmission of infectious diseases such as the West Nile virus [21]. Most of the plans identify the need for environmental management in order to protect biodiversity: Adelaide already has a relevant action plan which will be continued. Barcelona includes developing strategies for metropolitan green spaces and urban biodiversity, as well as protection of the coastal system’s biodiversity and functions. Cape Town plans to map, protect and rehabilitate ecosystems, and Santiago is planning to protect flora and the ecosystems of irrigation channels.

### 3.7. Risks to Vulnerable Populations

Vulnerability is the degree to which a system is susceptible and unable to cope with the adverse effects of climate change, including climate variability and extremes. Vulnerability is a function of the character, magnitude, and rate of climate change and variation to which a system is exposed, as well as the system’s sensitivity and adaptive capacity [85]. As we noted above, Mediterranean-climate urban populations are particularly vulnerable to climate change due to a number of synergetic reasons: cities contain concentrations of poor populations which are more susceptible to extreme weather events; Mediterranean climate regions are particularly vulnerable to projected climate change; and the urban design and infrastructure enhance extreme events such as heat, floods and droughts [19,20,23]. Therefore, we searched the urban climate adaptation plans for the inclusion of vulnerable populations. The plans we reviewed recognize this fact, and each one aims to reduce vulnerability by different means: Adelaide focuses on reducing exposure of vulnerable populations to extreme events, for example, by providing shelter. Barcelona is planning the mapping of vulnerable areas regarding health impacts under various climate scenarios. LA and Santiago plan to identify vulnerable populations, and also propose longer-term plans to distribute open spaces and greenery more equally. Cape Town, where the vulnerable population lives in much harsher conditions compared to the other cities we reviewed, is planning to take greater steps toward eliminating informal settlements and providing electrification in these communities.

### 3.8. Awareness Raising and Education

Another important adaptation measure is raising public awareness to protective behavior during extreme events, and to the health risks of climate change in the short- and long-term. Awareness and education components were included in all five plans surveyed, mostly detailing raising public awareness and building community resilience, as well as increasing knowledge among other stakeholders—e.g., local government, urban planners, civil society and the private sector. However, raising awareness and knowledge mostly remain general concepts, while the plans lack detailed means and specific objectives.

National governments can have a crucial role in the development and implementation of both mitigation and adaptation. Yet the local level is essential for protecting the public health of the city population from the emerging threats of the changing climate. We searched for Med-cities globally with climate adaptation plans, and found that many cities do not have such plans; and of those that do, their plans differ in scope (adaptation plans *vs.* adaptation as a component of mitigation plans), and that great gaps exist between plans in strategies and comprehensiveness regarding adaptation to health impacts. This resonates with a recent study of 200 medium-sized and large cities in Europe, which showed that 65% of them have mitigation plans while only 28% have adaptation plans, although these vary greatly in scope and ambition. In general, adaptation plans were found to be less concrete than mitigation plans, and their main foci were urban planning and development (approx. 75% of plans), water management (65%) and health aspects (60%). It is important to note that this study did not specify the different health aspects and adaptation measures [86].

## 4. Conclusions

Adaptation to climate change is a global challenge that supra-national institutions and individual countries are only now beginning to address in most regions of the world [9]. It is also a great challenge for local governance, where it remains a low priority due to busy agendas, limited resources, lack of professional knowledge, limited public support and, sometimes, limited decision-making authority [87,88,89,90]. For example, recent research found that climate action plans in the US fail to adequately protect health from climate change-related extreme heat [91] and, in Australia, urban planners, who are key professionals for adaptation at the city-level, are not aware of predicted climate change health impacts and adaptation measures [92].

Although there are differences between living standards, socio-economic levels and infrastructure in the five cities we examined, all of them are located in regions highly vulnerable to the impacts of climate change on the Mediterranean climate and therefore should deal with similar challenges. Some are contradictory phenomena, such as temperature extremes: intense heat waves in summer but also cold waves in winter, as well as floods and droughts, which may be more complicated than in more extreme environments. Four of the five cities are located by the sea and therefore have to deal with a rising sea level which may lead to floods, salinization of groundwater and negative impacts on water supply. While the populations of LA, Adelaide and Barcelona live in three of the 14 countries with the highest global socioeconomic rating, in severe weather conditions many residents may be at risk, particularly vulnerable populations. In those cities, 8%–11.2% of the population is unemployed and many others are below the poverty line (22% in LA and more than 28% in Barcelona). The elderly (21.6% of the population in Barcelona are above 65 years old) are also at high risk in conditions of severe heat or cold. Risks for health as a result of climate change are higher in cities such as Cape Town and Santiago, which are situated in less developed countries (see Table 1).

This paper examined the stated plans for adaptation to health impacts of climate change in five examples of Med-cities. We found that all five cities addressed the major “rising temperatures” driver, particularly heatwaves. However, while policy tools that target heatwaves are an established, simple and efficient measure (e.g., [93]), the plans we surveyed included fairly general notions of heat management. Urban design is another efficient measure to combat the rise of temperature in cities, but the cities studied used it randomly—one mentioned trees, another surface albedo, and a third city mentioned ventilation corridors, while all three of these examples of urban design are relevant to all five Med-cities we surveyed. Similarly, regarding the threat of flooding, four out of five plans addressed it, but one included sea level rise, stormwater discharge and green infrastructure; another focused on revitalizing blue infrastructure; and yet others focused on flood management and less on preventive infrastructure. Nevertheless, all five cities identified water supply and quality as a risk, and plan to reduce consumptions in different methods. All cities addressed vulnerable populations as a specific target population, and awareness and education as an overarching important component of adaptation, but with different foci.

We conclude that the five Med-cities with adaptation plans that we surveyed, and the relevant national adaptation plans and local health adaptation plans, address all the risks to health as identified in the local climate vulnerability assessments that were used [63,64,65,66,67,68,69,70,71,72,73]. However, the policy tools to adapt to these risks differ greatly and depend on the local context. It remains a future task to examine whether the measures identified in the adaptation plans are indeed the best ways to reduce risks to human health.

### Recommendations

In recent years, major international organizations have put forward recommendations for adaptation to climate change. For example, the United Nations International Strategy for Disaster Reduction launched the global campaign “Making Cities Resilient—My City is Getting Ready!” to promote increased understanding and commitment by cities and local governments to risk reduction and to build cities that are resilient to disasters and climate change. The overall target of the campaign is to get as many cities as possible committed to disaster risk reduction and to span a global network of engaged cities and municipalities of different sizes, characteristics, risk profiles, and locations that can help and learn from one another [85,94,95,96]. Based on these recommendations, and on the health risks in Mediterranean urban environments, we suggest that Med-cities may consider adopting the following fields depending on their local needs and vulnerability chalenges:

1. *Assessment of health risks*: conduct risk assessments in order to identify the main threats to urban public health in a Mediterranean climate, and to maintain up-to-date data on risks and vulnerabilities, transparent to the citizens.
2. *Extreme events management*: prepare comprehensive plans for extreme events, including early-warning systems. These plans should be cross-sectoral with a focus on vulnerable populations.
3. *Long-term adaptation*: go beyond short-term preparedness for extreme events, and prepare for the longer-term changing climate over a 20 to 50-year span, although this is a challenge due to much shorter political time frames. Specifically:
  - *Strategic urban planning*: based on risk assessments—building regulations, land-use planning, revising master plans.
  - *Micro-climate*: long-term adaptation for a hotter climate is a special opportunity for Med-cities, which can contribute to the wellbeing of the urban population throughout the hot Mediterranean climate. Cities should increase the amount of shaded public spaces, using trees both with wide canopies and other shading elements. The shaded areas should include public squares, playgrounds, bus stops, pavements on main streets and rest areas in parks [30].
  - *Social resilience*: strengthen communities and improve social networks.
  - *Physical resilience*: adapt water infrastructure for floods and drought, improve buildings, e.g., encourage homeowners to reduce risks of extreme weather.
4. *Risk communication*: raise awareness of the expected health risks, and promote opportunities for better understanding of adaptation health benefits (and co-benefits) among the general public, politicians, professionals in the municipality and city health system, local civil society organizations in the public sector, and schools.
5. *Collaborative governance*: design adaptation policies collaboratively across the different municipality departments, between the municipality and the public, local civil society organizations and local businesses, and across levels of government: local, national and international.
6. *Multi-level, global network governance*: to deal with health risks as a result of climate change, collaboration and coordination could be carried out not only at the county and/or state levels, but also with other cities dealing with similar challenges. An initial step toward collaboration between Med-cities has been taken recently with the establishment of the “Mediterranean City Climate Change Consortium (MC-4)” [97] that aims to coordinate efforts across political borders and disciplines to bring more resources and knowledge to building solutions for cities in the five Mediterranean-climate regions.
7. *Health systems*: build capacity and resilience in local public health agencies for short-term and long-term adaptation. The U.S. CDC developed a framework for Building Resilience against Climate Effects (BRACE) to facilitate the process in public health agencies [98].
8. *Ecosystems and natural buffers*: protect these resources to reduce the impact of climate change, including mitigation of floods and storms.
9. *Vector-, water- and food-borne diseases*: improve surveillance and control of climate-sensitive diseases.
10. *Research*: conduct local research to improve assessments of health risks, identification of locally-appropriate adaptation measures and evaluation of the implementation and outcomes of health-related climate action plans.

This current study examined in detail health aspects in climate change adaptation plans in five Med-cities. Although this is a preliminary step, our results and recommendations may serve as a basis for future preparedness in similar cities with a Mediterranean climate.

## Figures and Tables

**Table 1:** Descriptive data on the five selected Med-cities.

Variable	Adelaide	Barcelona	Cape Town	Los Angeles	Santiago
*Temperature (°C) and precipitation (mm) in January and July* [39]	Jan: max 29.2 °C; min 17.1 °C; 19.7 mm Jul: max 15.3 °C; min 7.5 °C; 76.9 mm	Jan: max 13.4 °C; min 4.4 °C; 41 mm Jul: max 27.5 °C; min 18.6 °C; 20 mm	Jan: max 26.1 °C; min 15.7 °C; 15 mm Jul: max 17.5 °C; min 7.0 °C; 82 mm	Jan: max 20.1 °C; min 8.8 °C; 79.2 mm Jul: max 28.4 °C; min 17.6 °C; 0.3 mm	Jan: max 29.7 °C; min 13 °C; 0.4 mm Jul: max 14.9 °C; min 3.9 °C; 86.6 mm
*Population size* [40,41,42,43,44]	21,618 (in 2012) and 1,225,235 in Greater Adelaide (2012)	1,602,386 (in 2014)	3,740,026 (in 2011)	3,928,864 (in 2014)	358,000 (in 2015)
*Socio-economic status* [45,46,47,48,49,50,51]	11.2% unemployed 11.5% below poverty line	10.2% unemployed, 28.1% at risk of poverty or exclusion	23.9% unemployed, No household income: 13.7% Less than 9600Rand (~$670): 6.7%	8.0% unemployed, 22% below poverty level	6.0% unemployed (national data), 14.4% below poverty line (national data)
*Population distribution* [42,45,52,53]	0–14: 17.7% 15–65: 66.9% 65+: 15.4% (in Greater Adelaide)	0–14: 12.6% 15–64: 65.8% 65+: 21.6%	0–14: 24.8% 15–64: 69.6% 65+: 5.5%	0–14: 19% 15–65: 70.5% 65+: 10.5%	0–14: 20.7% 15–59: 65.9% 60+: 13.4% (in Greater Santiago)
*World Bank economic rating of country (#/193 countries), 2014* [54]	12	14	33	1	42
*Human Develop. Index (HDI) rating of country (#/187 countries), 2014* [55]	2	27	118	5	41
*GDP of country ($), 2014* [56]	1.454 trillion	1.404 trillion	349.8 billion	17.42 trillion	258.1 billion
*Environ. Performance Index (EPI) of country (#), 2014* [57]	3	7	72	33	29
*Green space/number of trees per sq. km.* [58,59,60,61,62]	Park Lands = 9.3 km^2^	6.82 m^2^ green space/inhabitant in urban area 11.02 km^2^ urban green space	Over 50 km^2^ of accessible open space. 2.89 km^2^ district parks; 17.81 km^2^ community parks; 1.14 km^2^ biodiversity areas; 12.15 km^2^ greenbelts; 19.96 km^2^ road verges	Park space = 10% of city area	Green areas in Santiago Commune: 1.41–2.93 km^2^

**Table 2:** Climate Action Plans in the five Med-cities, analyzed according to the expected health impacts of climate change on city dwellers in local and national vulnerability assessments.

Mediterranean-Climate City	Adelaide, Australia	Barcelona, Spain	Cape Town, South Africa	Los Angeles, United States	Santiago, Chile
Risk Vulnerability assessments [63,64,65,66,67,68,69,70,71,72,73]	Adaptation Strategies [74,75]	Adaptation Strategies [66,76,77,78]	Adaptation Strategies [36,67,68,79,80]	Adaptation Strategies [70,71,81,82,83]	Adaptation Strategies [72,73]
**Rising Temperatures: Heat Waves, Heat Stress, Heat Island Effect** Identified as a local risk by all 5 cities	**Heatwave management:** Emergency management procedures; formalize extreme heat strategies. **Urban design:** Water sensitive urban design; increase vegetation in capital works projects. Urban Design strategies and approaches incorporating urban heat island mitigation measures (network of greenways, tree-lined streets, open spaces).	**Heatwave management: **Emergency program for extreme events; provide adaptation measures to protect exposed workers from increasing climate conditions (especially heat-related health risks). Early warning systems. **Urban design:** Include adaptation criteria (to heat island effect risks) in drafting new urban development plans; reform existing ones. Convert fleet of buses to hybrid/electrical as measure to combat heat island effect.	**Heatwave management:** Increase awareness of how to manage heat-related stress and other climate-related illnesses: Design and implement “Heat-Health” action plans, including plans in respect of emergency medical services. Nation-wide climate change and atmosphere monitoring systems/networks: Climate Change Response Monitoring and Evaluation System.	**Heatwave management: **Public Health agency to issue heat alerts, guidance to schools, ensure appropriate resources (*i.e.*, cooling centers) available to public. Establish, improve and maintain mechanisms for robust rapid surveillance of environmental conditions, climate-related illness, vulnerabilities, protective factors and adaptive capacities. **Urban design:** Million Trees Initiative to increase tree canopy throughout the city.	**Heatwave management:** Monitoring system WebGIS, Adapt monitoring systems and emergency plans by including any climate change related health effects in risk management practices. **Urban design:** Green standards in new development projects; protection of ventilation corridors. More green spaces to reduce heat island effect.
**Heavy Rainfall and Flooding** Identified as a local risk by all 5 cities	**Infrastructure:** Design public spaces to assist in adapting to climate change; incorporation of green infrastructure into the renewal of the City. Adaptation measures and protection from predicted sea level rise in Development Plans. Protect existing infrastructure (stormwater discharge management).	**Flood management:** Risk mapping, prevention plan, emergency plan, action plan for flood zones. Implementation of monitoring systems and systematic data input.	**Flood management:** Climate Adaptation Plan of Action (CAPA): sea-level rise risk assessment and economic modeling; Coastal Protection Zone By-law; stormwater management responses to more intense rainfall, sea-level rise and storm surges. Nation-wide climate change and atmosphere monitoring systems/networks: Climate Change Response Monitoring and Evaluation System.	**Emergency management: **Develop comprehensive plans to prepare for climate change impacts, *i.e.*, increased drought, wildfires, sea level rise, and public health impacts. Climate forecasting, sea level rise; vulnerability and risk assessments.	**Flood management:** Monitoring system WebGIS. Adapt monitoring systems and emergency plans by including any climate change related health effects in risk management practices. **Infrastructure:** Revitalizing existing water flow networks (river irrigation channels); permeable pavements. More green spaces to reduce flooding exposure.
**Fresh water supply and quality** Identified as a local risk by all 5 cities	**Water Quality:** Continue implementation of Biodiversity and Water Quality Action Plan.	**Water management: **Drought control. Update inventory of wells, recover wells and aquifers dismissed as resource for irrigation and gardening water, better treatment/use of wastewater; improve water use efficiency.	**Water management:** Reduce water demand, adopt an integrated water management approach, conduct long-term monitoring of selected hydro-meteorological parameters.	**Water management:** Water conservation and recycling, reduced water consumption, implement water and wastewater integrated resources plan, including capture and reuse of stormwater. Maintain and upgrade water accessibility information.	**Water management:** Reduce agricultural water demands through efficient irrigation technologies—sustainable water balance between availability and demand, reduce exploitation of groundwater resources, increase environmental flows.
**Air Quality** Identified as a local risk by Barcelona, Cape Town and Los Angeles assessments		**Reduce emissions:** Promote use of biofuels; reduce GHG emissions and suspended particle matter. Urban design: Establish spaces free of motorized vehicles; create a more equitable city.	**Reduce emissions:** A key strategy to reduce the brown haze. Reduce ambient PM, ozone, and sulphur dioxide concentrations by legislative and other measures. Nation-wide climate change and atmosphere monitoring systems/networks: Climate Change Response Monitoring and Evaluation System.	**Reduce emissions: **Lower impact of carbon intensity of transportation sector (directly related to smog and toxic air pollutants).	**Urban design:** More green spaces to improve air quality.
**Water-Borne Disease, Food-Borne Disease, Vector-Borne Disease** Identified as a local risk by Barcelona, Cape Town, Los Angeles and Santiago assessments		Surveillance programs and control programs for vector-borne diseases.	Improved climate-sensitive disease surveillance and control, safe water and improved sanitation. Increased support for public health facilities in dealing with diarrhoea and dehydration; improved sanitation in informal settlements. Improve the bio-safety of the current malaria control strategy; strengthen the awareness program on malaria and cholera outbreaks.	Develop existing environmental contaminant biomonitoring. Track data on environmental conditions and associated diseases related to climate change.	Create and develop capacities to address the potential introduction of yellow fever, dengue fever, malaria and vectors such as aedes and anopheles mosquitoes.
**Loss of Biodiversity, Functioning Ecosystems** None of the cities identified this as a risk	**Environmental management:** Maintenance of vegetation health, protection of biodiversity; continued implementation of Biodiversity and Water Quality Action Plan.	**Environmental management:** Develop strategy for metropolitan green spaces and urban biodiversity. Gradual adaptation of vegetation with species adapted to climatic conditions (low water consumption, more fire resistant). Protect/conserve coastal system biodiversity to maintain healthy ecosystem functions (*i.e.*, seagrass beds that act as a carbon sink, help reduce the erosion effect of coastal dynamics, and stabilize beaches). Map vulnerability of biodiversity, consolidate ecological monitoring networks.	**Environmental management:** Protection, management and rehabilitation of functioning ecosystems. Mapping and identification of the functioning ecosystems that must be protected and managed. Monitoring climate change impacts.		**Environmental management: **Protection of urban biodiversity (trees, gardens). Revitalizing existing water flow networks (river irrigation channels) and their ecosystem functions.
**Overarching concept: Risks to Vulnerable Populations**	**Strategic plan:** Adelaide City Council Emergency Plan 2012–2016 completed. Strategy covers extreme weather events (including heat waves and flooding). Adelaide Central Bus station established as refuge for the community during extreme heat conditions. Continued coordination with Street to Home service providers to monitor and assist rough sleepers during extreme heat events.	Assessment of the effects of climate change on health and mapping of vulnerable areas under different climate scenarios.	**Urban design and infrastructure:** Improve management and eventual elimination of informal settlements, electrification and improved public transport in urban areas to reduce local pollution levels and improve the quality of life of the poor, and reduce their vulnerability to extreme climate events.	**Urban design: **Create a more equitable distribution of open space, greenery, and recreational opportunities. Develop preparedness and response plans to identify vulnerable populations in Los Angeles County.	**Urban design: **Halt decline of existing green spaces and increase new ones in urbanization projects and vulnerable zones; create more urban green spaces with public participation, increase public access and use of green spaces. Identify vulnerable areas or those with the greatest health risks due to different factors; consider the affected population.
**Overarching policy mechanism: Awareness raising and education**	Build community knowledge, capacity and resilience in adapting to climate change; communication and awareness campaigns (research, stakeholder forums).	Increase knowledge and awareness of climate change and effects among municipal agents and wider public. Public participation campaigns on climate change and health.	The Climate Smart Cape Town Campaign promotes climate change literacy and awareness among residents and decision-makers. The Cape Town Climate Change Coalition includes businesses, NGOs, academia, and provincial and local government. “Communication, Education and Public Awareness project (CEPA). Develop public awareness campaigns on health risks of high temperatures and appropriate responses.	Improve the public’s capacity to respond to an emergency. Develop an educational campaign to increase public awareness of the health impacts of climate change, and educate staff.	Education/awareness campaign for greater public; educate/train public officials responsible for urban planning in climate change and its impacts. Strengthen the capabilities of health personnel to address prevention and care of adverse effects caused by climate change. Interact with other sectors in order to identify effects of climate change on the health of the population.
